# Fiber-Optic Localized Surface Plasmon Resonance Sensors Based on Nanomaterials

**DOI:** 10.3390/s21030819

**Published:** 2021-01-26

**Authors:** Seunghun Lee, Hyerin Song, Heesang Ahn, Seungchul Kim, Jong-ryul Choi, Kyujung Kim

**Affiliations:** 1 Departments of Congo-Mechatronics Engineering, Pusan National University, Busan 46241, Korea; s.lee@pusan.ac.kr (S.L.); rin8520@gmail.com (H.S.); ahn3890@gmail.com (H.A.); s.kim@pusan.ac.kr (S.K.); 2 Department of Optics and Mechatronics Engineering, Pusan National University, Busan 46241, Korea; 3 Medical Device Development Center, Daegu-Gyeongbuk Medical Innovation Foundation (DGMIF), Daegu 41061, Korea

**Keywords:** fiber-optic sensor, surface plasmon resonance (SPR), localized surface plasmon resonance (LSPR), nanomaterials, nanostructures, nanoparticles

## Abstract

Applying fiber-optics on surface plasmon resonance (SPR) sensors is aimed at practical usability over conventional SPR sensors. Recently, field localization techniques using nanostructures or nanoparticles have been investigated on optical fibers for further sensitivity enhancement and significant target selectivity. In this review article, we explored varied recent research approaches of fiber-optics based localized surface plasmon resonance (LSPR) sensors. The article contains interesting experimental results using fiber-optic LSPR sensors for three different application categories: (1) chemical reactions measurements, (2) physical properties measurements, and (3) biological events monitoring. In addition, novel techniques which can create synergy combined with fiber-optic LSPR sensors were introduced. The review article suggests fiber-optic LSPR sensors have lots of potential for measurements of varied targets with high sensitivity. Moreover, the previous results show that the sensitivity enhancements which can be applied with creative varied plasmonic nanomaterials make it possible to detect minute changes including quick chemical reactions and tiny molecular activities.

## 1. Introduction

Optical sensors are defined as devices and instruments that observe minute changes in the optical signal (absorbance, reflectivity, refractivity, etc.) to detect the characteristics of a sample or changes to the measurement target [1,2]. Optical sensors have been actively employed in various fields of education, research and industry due to advantages of non-destructivity for samples, high-sensitivity, and appropriate selectivity. In particular, optical sensors based on surface plasmon resonance (SPR) is based on a phenomenon of resonant oscillation of electrons between a metallic substrate and a dielectric superstrate stimulated by incident light [3,4]. The SPR sensors as a high-performance sensing technique that can detect fine refractive index changed by differences in molecules or smaller unit of matter have been commercially succeed in the fields of physics, chemistry, biotechnology, and medicine. Moreover, localization of surface plasmon resonance using nanotechnology has been widely studied for further sensitivity enhancements [5].

As the optical sensors are used in a variety of ways, the issue of precise, reliability and ease-to-use of optical sensors become more important. To increase mobility, accessibility to samples, and practical usability in optical sensors, optical elements and techniques are needed to efficiently transmit and receive light while making the sensor small. An optical fiber, that can reflect light from the inside and move the light where it wants to be, is one of the adequate optical elements that meets the conditions. A multi-core fiber bundle, consisting of several optical fibers, also has a small diameter of around 1 to 2 mm, and optical fibers can be connected to most of light sources and detectors using fiber-optic couplers. For these reasons, an implementation of optical sensing techniques in the optical fiber has been employed to ensure practicality regardless of the environment [6,7,8]. For instance, a fiber-optic temperature sensor with phosphorescent materials at the end of the fiber has been employed to measure temperatures of a sample inside a magnetic resonance imaging instrument that has a high magnetic field so that light sources and detectors cannot enter [9]. Research that combines optical sensors based on SPR with optical fibers has been applied to the development of optical sensors with securing both practical usability and highly improved sensitivity [10,11].

Many review papers on fiber-optic sensors have been investigated. Most of the previous research showed the general fiber-optic sensors and their applications with varied materials and fabrication process which includes much wider variety of categories. Some of the review articles emphasized the biological aspect of the sensor with diagnosis and clinical applications [12,13,14]. Some of the articles describes the trends and future roadmap of the sensors [15]. On the other hand, there are few research reviews exist about the fiber-optic sensor which was described as a focal point for localized surface plasmon resonance and improvement of its sensitivity. Thus, in this article, we described the contents from a different perspective. The principles of fiber-optic SPR sensors and the recent research of fiber-optic localized SPR (LSPR) sensors are included. Moreover, the key research techniques using nanomaterials for fiber-optic LSPR sensors are investigated. The most important difference and highlight point compared to the previous review papers is to focus on the research not only the concrete principles or applications of the fiber-optic sensor itself but investigating the special cases by using localized surface plasmon resonance to the fiber-optic sensors for the further sensitivity enhancement of a sensor. In particular, we focused more on recent studies in applications of fiber-optic LSPR sensors using nanostructures or nanoparticles on fiber-optic sensors [12,13,14,15,16,17,18,19,20,21], and interesting practical applications using fiber-optic LSPR sensors are explored with three different categories: (1) chemical reactions measurements, (2) physical properties measurements, and (3) biological events monitoring. The applications in this article are chosen mainly according to the LSPR based fiber-optics sensor with the various types of nanoplasmonic materials; spherical metal nanoparticles, nanodots, nanodisks, nanomushroom, nanoposts and nanoholes in a metal film. These results show a great possibility of fiber-optic LSPR sensor that it can be used in practical applications for varied analyte detection by a superb sensing performance. In addition, a brief explanation and prospect for commercial use of fiber-optic sensors and subsequent studies to improve their limitations were described in the concluding remarks.

## 2. Fundamental Principles of Fiber-Optic Localized Surface Plasmon Resonance Sensors

### 2.1. Fiber-Optic Plasmonic Sensors

**Figure 1:**
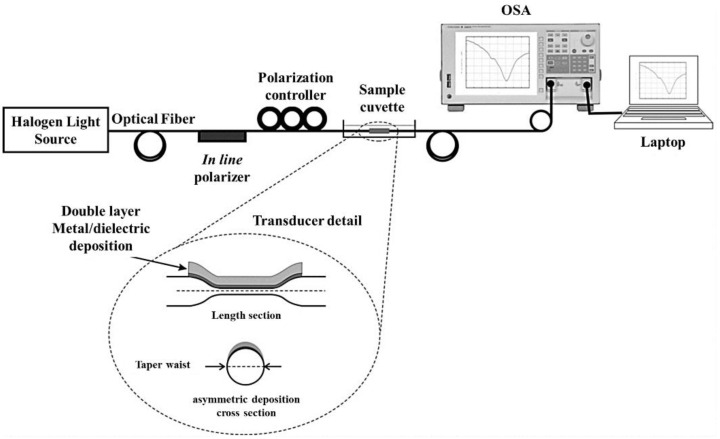
Scheme of the experimental setup and detailed view of the fabricated transducers. The figure in Esteban et al. [32] is reprinted with permission from Elsevier.

As fiber-optic sensors can be used to convert physical quantities such as pressure, temperature, and flow rate into measurable quantities with an optical-transmission-waveguide-based complex device system, conventional optical fibers are used for many different applications ranging from biomaterial imaging to monitoring of hazardous environmental toxicants. Accumulating data through optical sensors by means of photonic technologies is a relatively simple method to analyze and acquire scientific information of interest. With increasing interest in sensor technology, the development of highly selective and sensitive optical sensors for medical diagnosis and monitoring of analytes has grown rapidly over the past decade. Recent advances in fiber optics have boosted the demand for optical sensors in various fields as fiber-optic-based sensors provide various types of information by measuring the intensity, frequency, phase, and polarization of light [22,23,24,25]. Thus, fiber-optic sensors have been widely used in measuring physical [26] and chemical [27] quantities, especially in the monitoring of pressure [28], refractive index of analytes [29], and concentration of chemical species [30]. Fiber-optic sensors provide excellent performance in many of these applications. However, there are some limitations. Owing to the cost and complexity of these devices, it is difficult to adopt fiber-optic sensors in specialized industrial and clinical applications as well as experimental studies that require low production costs and high sensitivity. In the last few years, a new type of fiber-optic sensor called plasmonic fiber-optic sensors has emerged. Plasmonic fiber-optic sensors make use of the plasmonic effect, which is based on absorption intensity measurements. A series of performance-enhanced fiber-optic plasmonic sensors has generated considerable attention in the research field. These performance-enhancement techniques can be divided into two types of methods depending on the geometry of the modified fibers and on the contact points on the fiber at which the plasmonic nanostructures are applied. In the first method, part of the fiber cladding is removed to deposit the plasmonic substrate. In the second method, plasmonic substrates are implanted at the end of the optical fiber. The first method, in which localized surface plasmon resonance (LSPR) is excited by removing a part of the cladding is based on evanescent absorption of light by total internal reflections. Dwivedi et al. proposed a cladding-removed fiber-optic sensor for glucose detection, for which the sensor performance depended on the core diameter and sensing region [31]. The sensitivity, signal-to-noise ratio (SNR), and resolution of the sensor were evaluated, and it was found that to obtain the best sensing performance, a large core diameter and a small sensing region were required. Esteban et al. investigated indium nitride dielectric overlay of optical fibers for highly sensitive surface-plasmon-resonance (SPR) sensing as described in Figure 1 [32]. They demonstrated the suitability of the use of indium nitride for the development of SPR sensors. It was proved that the performance of the sensor depended on the InN thickness, and the sensor could achieve sensitivity as high as 11,800 nm/RIU.

**Figure 2:**
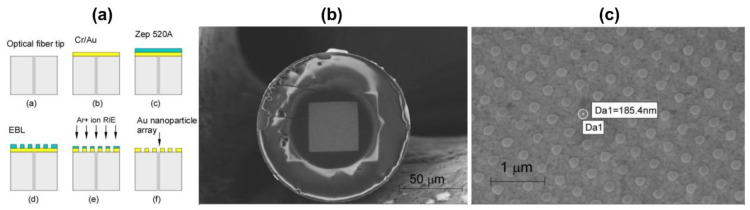
Fabrication process for gold nanodots arrays on the optical fiber tip (**a**), scanning electron micrograph of the gold nanodot array on the optical fiber (**b**) and gold nanodot array on the optical fiber facet (**c**). The figure in Lin et al. [33] is reprinted with permission from Multidisciplinary Digital Publishing Institute (MDPI).

Another application which makes use of the modification of the fiber end has been developed. This method utilizes the nano plasmonic transmission difference between the sensors. As shown in Figure 2, Lindquist et al. [33] investigated e-beam patterned gold nanodot arrays on optical fiber tips for LSPR sensing. LSPR of the gold nanodot arrays patterned on optical fiber tips has been used for refractive index sensing with a high sensitivity of 196 nm/RIU, and this sensor was used to detect streptavidin with a limit of detection of 6 pM.

### 2.2. Localized Surface Plasmon Resonance (LSPR)

LSPR is defined as a resonance phenomenon caused by surface plasmons. Plasmons are electron vibrations generated by the interaction of conductive electrons on metallic substrates with light of a specific wavelength or an oscillating electric field. LSPR is generated when small metallic nanostructures or nanoparticles are irradiated with optical wavelengths larger than the size of the nanoparticle. SPR is influenced by the incident wavelength of light and the properties of the metal and dielectric substrates. LSPR is also affected by the size, arrangement, and shape of the integrated nanostructures or nanoparticles.

Generally, LSPR based on well-designed nanostructures or nanoparticles under certain conditions induces higher localized electrical field enhancements than that by SPR without the nanomaterials. Hence, LSPR plasmonic sensors show improved sensitivity. For instance, 2D arrays of metallic subwavelength nanoapertures with a period of 200 nm and a diameter of 80 nm offered a sensitivity 2.54 times higher than that of a prism-type SPR sensor for the detection of avian influenza DNA hybridization [34]. Through sophisticated nanostructure fabrication and nanoparticle assembly, advanced LSPR sensors that provide further sensitivity enhancement have been developed. For example, a 3D nanogap array, which was fabricated by angled evaporation and development, showed a sensitivity improvement of more than 1000 times for the detection of surface bio affinity interactions [35]. Bow-tie optical antennas can generate highly enhanced plasmonic fields. LSPR sensors using the bow-tie nanostructures offer improved sensitivity for measuring data of specific chemical and biological interactions [36,37,38].

### 2.3. Integration of Localized Surface Plasmon Resonance to Fiber-Optic Plasmonic Sensors

**Figure 3:**
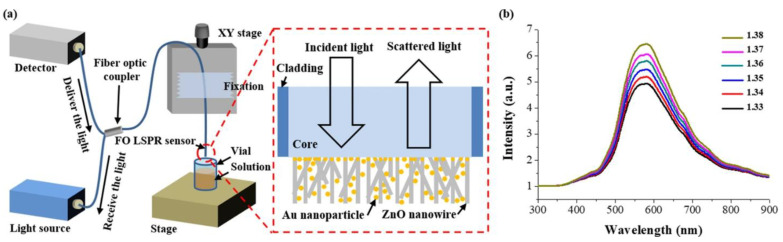
Schematic of the optical system and the LSPR spectra measured using the sensor proposed in Kim et al. [47]: (**a**) Measurement set-up based on the optical fiber and (**b**) spectra measured using 3D FO-LSPR sensor for solutions of different refractive indices. The figure in Kim et al. [47] is reprinted with permission from Nature publishing Group (NPG).

As the recent research proved that localized surface plasmon resonance is well integrated into fiber-optic sensors, various solutions have been suggested for the performance enhancement of optical-fiber-based sensors for different applications. Those applications are briefly classified into three different cases; a method based on evanescent wave absorption [39,40], evanescent wave light scattering [41,42], fiber grating [43,44] and photonic crystal fiber [45,46]. The most common method to improve the sensitivity of the sensor is to use plasmonic nanostructures on the fiber-optic sensor for LSPR generation. Various solutions have been suggested for the performance enhancement of optical-fiber-based sensors for different applications. The most common method to improve the sensitivity of the sensor is to use plasmonic nanostructures on the fiber-optic sensor for LSPR generation. The most commonly used materials for plasmonic enhancement are silver, gold, and copper. Moreover, metallic nanoparticles with specific morphologies have been found to enhance the performance of the sensor in terms of sensitivity, resolution, stability, and effective range of detection. As shown in Figure 3, Kim et al. [47] developed plasmonic biosensors with ZnO nanowires decorated by gold nanoparticles. The 3D ZnO nanowires grown on the surface of optical fibers were deposited with immobilized gold nanoparticles to improve the sensitivity of optical fiber-based LSPR sensors. The 3D fiber-optic LSPR sensor showed a sensitivity enhancement of approximately 171% for bulk refractive index changes when compared to 2D fiber-optic LSPR sensors. This sensitivity enhancement is ascribed to the morphology difference between the gold nanoparticles randomly distributed in a 3D volume and a monolayer attached on the optical fiber. The sensors were used to measure various concentrations of a prostate-specific antigen biomarker, and the limits of detection of the 2D and 3D fiber-optic LSPR sensors were found to be 2.06 pg/mL and 0.51 and pg/mL, respectively.

**Figure 4:**
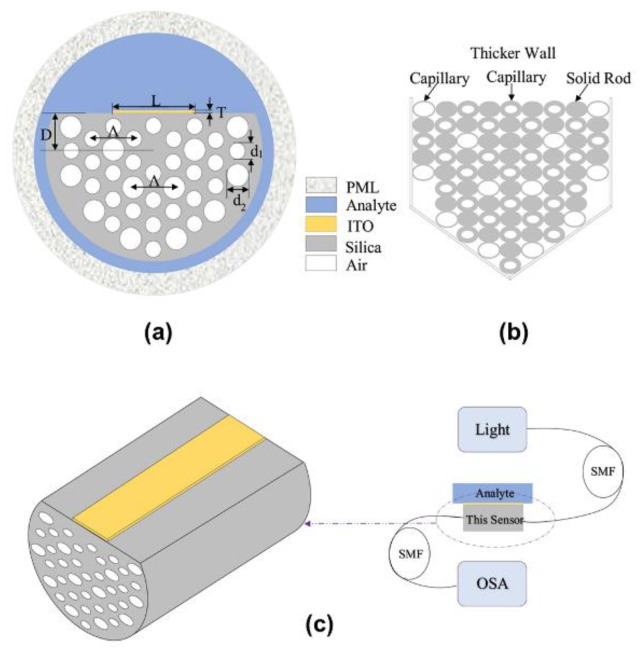
(**a**) Cross-section schematic of the sensor, (**b**) stacked structure of the sensor, and (**c**) 3D illustration of the sensor. The figure in Liu et al. [48] is reprinted with permission from Elsevier.

Another fascinating material which has been used in fiber-optic sensors is indium tin oxide (ITO). Liu et al. [48] reported a surface plasmon resonance sensor based on a D-shape crystal fiber as described in Figure 4. As it expands the operating wavelength range of the sensor to the infrared region, ITO was chosen as the plasmonic material. ITO can be used for low refractive index sensing. The D-shape photonic crystal fiber prepared by side polishing was coated with ITO films of various thicknesses adjacent to the core to enhance the sensing performance by promoting the strong interaction of the evanescent waves with the analytes. The ITO coated sensor has a maximum spectral sensitivity of 15,000 nm/RIU and has a resolution of $6.67\times 10^{-6}$ RIU. The operating range of the sensor the infrared region is from 1200 nm to 2250 nm for low RIs from 1.22 to 1.33.

## 3. Recent Studies of Fiber-Optic Localized Surface Plasmon Resonance Sensors Using Nanomaterials

As the fiber-optic sensor technology has experienced rapid growth in the past few decades, a significant research in fiber-optic sensor has been investigated due to the tremendous attention by its several inherent advantages [40,43,49] Those pioneering works comprise underlying ideas which are the basis of modern optical sensing technologies. Meriaudeau et al. [50] proposed significant research achievements which based on gold island surface plasmon excitation of the fiber-optic chemical sensor to measure the optical index of adsorbates. Moreover, the novel concepts of the sensors which make use of evanescent wave were reported to improve the detection limit of the sensor with plasmonic particles. Cheng et al. [39] proposed refractometry sensor with the self-assembled gold colloids on the optical fiber. Kajikawa et al. [51] reported a highly sensitive micrometer size optical fiber affinity biosensor without any labeling process. And the subsequent research based on the previous works were investigated. The recent concepts and advantages of fiber-optic LSPR sensors described in Section 2 which have been employed in the development of highly sensitive practical sensors for measuring physical properties and detecting specific chemicals. In this section, fiber-optic LSPR sensors used for three different applications are discussed: chemical sensing, measurement of physical properties, and biological and biomedical sensing. Various nanoplasmonic materials have been used in recent studies to establish high-performance fiber-optic LSPR sensors: a spherical metal nanoparticle [52], nanoholes in a metal film [53], carbon nanotubes [54], graphene and graphene oxide nanosheets [55], metal nanodots [56], metal nanodisks [57], metal nanomushroom [58] and metal nanorods [59].

### 3.1. Chemical Fiber-Optic Localized Surface Plasmon Resonance Sensors Based on Nanomaterials

Fiber-optics-based chemical sensors are used for detecting a specific chemical analyte. The sensor transforms various chemical properties into an optical property that can be detected as an electrical signal. For improving the detection of low concentrations of small target molecules, a plasmonic effect has been employed in fiber-optic-based chemical sensors by using nanomaterials. Chemical fiber-optic sensors are commonly used for measuring data on liquid or gaseous phase of environmental changes, for instance, detecting ions diluted in solution or in gas phase, along with the corresponding pH level.

**Figure 5:**
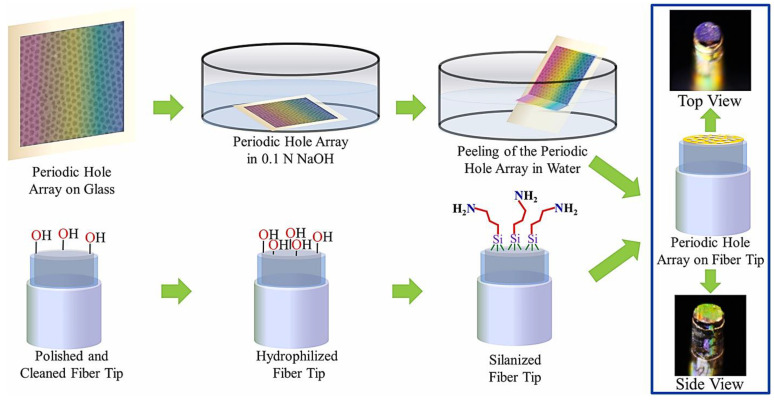
Process used to prepare fiber-optic plasmonic sensors. A periodic hole array in a gold film is fabricated on a flat glass substrate using solely chemical methods. Subsequently, this hole array is lifted off from the substrate surface by immersing it in a basic solution. The hole array film detaches from the substrate surface and starts floating on the water surface. The periodic hole array in a gold film can be picked up with an appropriately functionalized optical fiber tip. Photographs of the resulting fiber-optic plasmonic sensors are shown on the right. The figure in Polley et al. [53] is reprinted with permission from Elsevier.

Scherino et al. [52] fabricated a nanohole pattern at the tip of the fiber and deposited a stimuli-responsive polymer for detecting and measuring pH changes. The metallic nanomaterials at the tip were designed to improve the sensitivity of the fiber-optic sensor to superficial environmental changes. The poly(N-isopropylacrylamide-co-acrylic acid) microgel(PNIPAm-co-AAc MG) deposited on the nanopatterned fiber tip expands/contracts in response to pH. This method is an indirect method of measurement as the different concentrations of ions affect the optical properties of the stimuli-responsive polymer that is attached to the sensing area. Thus, the fiber-optic sensor converts chemical properties of the solution into optical properties. Polley et al. [53] proposed periodic hole arrays in gold films on optical fiber tip for plasmonic sensing platforms which are able to be integrated with optical fibers and can be used as sensitive biosensor platforms as shown in the schematic in Figure 5.

**Figure 6:**
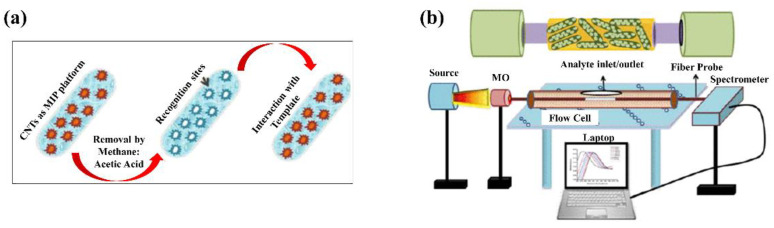
A schematic illustration of fabrication and testing process for fiber-optic sensors; (**a**) formation of surface imprinted sites on carbon nanotubes (CNTs), and (**b**) experimental set-up for dopamine sensing. An optical fiber of core diameter 600 um and NA 0.4 is uncladded from the middle 1 cm length and coated with 40 nm silver film for excitation of SPR. Surface imprinted CNTs suspensions were prepared by vinyl group and molecular imprinted polymers (MIPs) for sensing probe. This entrapment of dopamine molecules in the imprinted cavities on CNTs results in increase in the refractive index of CNTs layer. The figure in Pathak and Gupta [54] is reprinted with permission from Institute of Electrical and Electronics Engineers (IEEE).

A. Pathak and B. D. Gupta proposed a fiber-optic dopamine sensor utilizing an imprinted carbon nanotube platform and the surface plasmon resonance technique as described in Figure 6 [54]. The sensor possesses a dynamic range of 0–150 μM and a detection limit of 2.1 μM.

**Figure 7:**
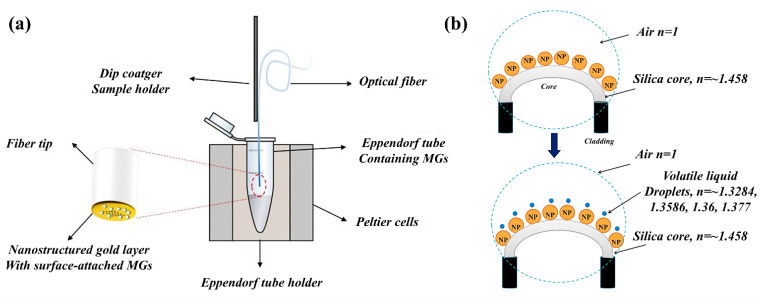
(**a**) Schematic of the nanopatterned fiber-optic sensor. The nanohole array was patterned on the tip of the fiber. The stimuli-responsive polymer is deposited on the nanopatterned area as shown in the figure on the right. The figure in Giaquinto et al. [62] is reprinted with permission from Multidisciplinary Digital Publishing Institute (MDPI). (**b**) Schematic of LSPR U-bent fiber-optic gasoline sensor using noble metallic nanoparticles coated on the U-bent fiber core. The figure in Saikia et al. [60] is reprinted with permission from Elsevier.

Researchers have studied the effect of the geometrical properties of the nanostructures on the detection properties of the fiber for enhancing the performance of LSPR fiber-optic chemical sensors. Several studies have explored fiber-optic sensors with a U-bent shape. Paul et al. [60] used two different types of noble metal-based LSPR U-bent fiber-optic sensors for detecting vapors of volatile liquid. The nanoparticles exploited for enhancing sensing performance were coated on the core region, as shown in Figure 7b. The sensitivity and plasmonic response of the LSPR U-bent fiber-optic sensor using different types of noble metallic nanoparticles were studied and compared. Saikia et al. [61] used a silver nanostructured and tapered U-bent fiber-optic probe as a pH sensor. The effects of factors such as tapering and the presence of the nanostructures on the sensitivity of U-bent fiber-optic probe were investigated. Interestingly, the tapered U-bent fiber contributed to an improvement in sensor sensitivity. Furthermore, it was found that the nanostructures on the U-bent fibers also contribute significantly to sensitivity enhancement.

### 3.2. Fiber-Optic Localized Surface Plasmon Resonance Sensors to Measure Physical Properties

Sensors that provide information about the physical properties of the specimen are important and actively utilized as they can obtain the most basic and intuitive information. Representative physical properties include temperature and pressure on contact in the internal/external regions of the specimen. Different methods have been employed for measuring temperature or pressure using fiber-optic probes [63,64,65,66]. Several research groups have investigated fiber-optic LSPR sensors for the measurement of physical properties, especially temperature, with further enhancements in sensitivity and precision.

**Figure 8:**
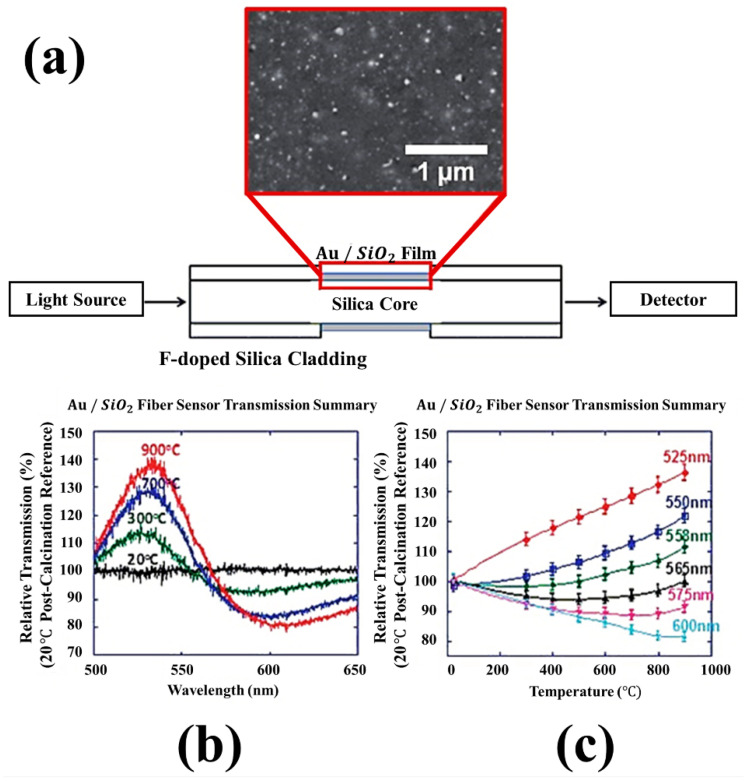
(**a**) Schematic of a fiber-optic temperature and gas sensor that is capable of operating in extreme temperature conditions. The inset figure shows the scanning electron microscopic image of Au/SiO_2_ nanocomposites fabricated on the fiber-optic temperature and gas sensor. (**b**) Transmission spectra measured by the fiber-optic temperature and gas sensor at 20, 300, 700, and 900 °C. (**c**) Relationship between the temperature and relative transmission according to wavelength of the light source introduced in the fiber-optic sensor. The figure in Ohodnicki et al. [69] is reprinted with permission from Royal Society of Chemistry (RSC).

Srivastava et al. [67] proposed a fiber-optic LSPR sensor for temperature measurement. This fiber-optic LSPR sensor was coated with a dielectric sensing medium, and gold nanoparticles with a diameter of 5 nm were deposited in a partial area from which the cladding was removed. LSPR in the coated region was employed for the detection of temperature with high sensitivity. Simulation studies were employed to explore the most optimal dielectric material in the sensing medium for realizing a fiber-optic LSPR sensor that is the most responsive to temperature changes. Algorri et al. [68] investigated a fiber-optic LSPR temperature sensor using metallic/semiconductor nanoparticles doped on a liquid crystal layer. Ohodnicki et al. [69] developed a fiber-optic temperature and gas sensor that could be used in extreme temperature conditions, as illustrated in Figure 8. This fiber-optic sensor was developed by fabricating temperature- and gas-sensitive gold/silicon dioxide (SiO_2_) nanocomposite films and depositing them on the fiber. The film was coated on the optical fiber in regions where the cladding was removed. This fiber-optic sensor, which measures the shift of the LSPR absorption peak, could operate at temperatures over 900 °C, and it offered highly sensitive measurement of concentration changes for three gases (H_2_, O_2_, and CO) at 850 °C. The proposed fiber-optic temperature and gas sensor based on LSPR of nanocomposites has various applications as it can provide physical and chemical information in harsh industrial environments.

### 3.3. Biological Applications of Fiber-Optic Localized Surface Plasmon Resonance Sensors

A biological sensor is a device that can detect biological components by observing biological changes such as coupling, reactions, and reconstruction. Biological components of interest include antibodies, nucleic acids, and enzymes. Among the various signal detection and information acquisition modalities in biological sensors, optical approaches have been actively studied as they can offer high specificity, high sensitivity, feasibility of direct/real-time measurements, and scope for miniaturization [70,71]. In particular, SPR-based optical biological sensors have been researched [72,73,74,75] and commercialized in several enterprises [76,77,78] as they can measure fine variations in refractive indices with high sensitivity. Studies of biological sensors using nanomaterial-based LSPR to further improve sensitivity have also been actively pursued [34,79,80,81,82,83]. A series of studies have been conducted using LSPR biological sensors, which are connected to optical fibers. These sensors are capable of acquiring valuable biological information with very small amounts of samples.

**Figure 9:**
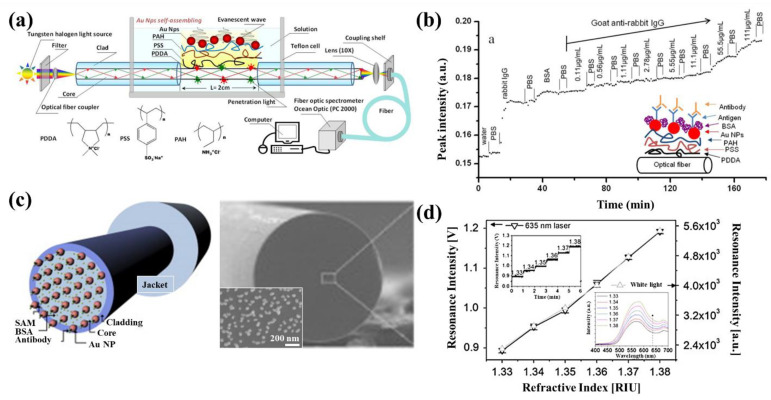
Fiber-optic localized surface plasmon resonance (LSPR) sensors for the detection of antibody–antigen interactions. (**a**) Schematic of a fiber-optic LSPR sensor with a gold nanoparticle assembly formed on a polyelectrolyte multilayer and (**b**) transient sensing of goat anti-rabbit IgG using the fiber-optic LSPR sensor. Figure 9a,b are from Shao et al [86], reprinted with permission from Multidisciplinary Digital Publishing Institute (MDPI). (**c**) Schematic and scanning electron microscopic image of a fiber-optic LSPR sensor for the detection of antibody–antigen interactions of interferon-gamma (IFN-γ). (**d**) The differences in the resonance signal of the fiber-optic LSPR sensor with varying concentrations of IFN-γ. The reprint of the figures in Jeong et al. [87] is permitted by Elsevier.

An antigen-antibody interaction is a biochemical phenomenon in which the antigen causing the immune response and the corresponding antibodies react with each other [84,85]. Fiber-optic LSPR biological sensors that utilize antibodies to detect specific antigens with high sensitivity have been developed. These sensors which can measure changes in refractive index from corresponding phenomena in real time are being actively studied for use in disease diagnosis and detection of dangerous biological materials. Lin et al. investigated gold nanodot arrays integrated on a fiber-optic tip for LSPR-based biochemical measurements [33]. The gold nanodots with 185 nm diameter and 55 nm height were fabricated on a square area of 40 μm × 40 μm on the optical fiber tip by electron beam lithography. This fiber-optic LSPR sensor using the gold nanodots had a sensitivity of 196 nm/RIU and was used to detect biotin-streptavidin reactions. The sensor had a limit of detection of 6 pM for streptavidin. Shao et al. developed a fiber-optic LSPR sensor with a gold nanoparticle assembly formed on a polyelectrolyte multilayer, which was deposited on the side of an optical fiber as shown in Figure 9a,b [86]. The gold nanoparticle assembly layer was coated on the tri-layer of polyelectrolyte structures with high efficiency (Figure 9a). The fiber-optic LSPR sensor with the gold nanoparticle layer offered improved sensitivity in the sensing of goat anti-rabbit immunoglobulin (IgG). Jeong et al. [87] investigated a fiber-optic LSPR sensor for antibody–antigen interactions of interferon-gamma (IFN-γ). The fiber-optic sensor was fabricated by applying gold nanoparticles on the end plane of an optical fiber as described in Figure 9c. A self-assembled monolayer (SAM) was coated on the end surface of the core (with a diameter of 105 μm) of the optical fiber by dipping the fiber-optic sensor in a colloidal solution of gold nanoparticles. The sensitivity of the sensor with varying refractive indices and the measurement of antibody–antigen interactions of IFN-γ are shown in Figure 9d. These results prove that the fabricated fiber-optic LSPR sensor can be applied as a biosensor that can detect various biological molecules and interactions with enhanced sensitivity and selectivity. Lin et al. developed a fiber-optic biochemical LSPR sensor using a tapered fiber and immobilized gold nanoparticles [88]. A preliminary study of measuring the change in refractive index in a flow cell surrounding the tapered fiber-optic LSPR sensor confirmed that the sensor had a sensitivity of 3.2 × 10^−5^ RIU. The tapered fiber-optic sensor was functionalized with *N*-(2,4-dinitrophenyl)-6-aminohexanoic acid (DNP) for anti-DNP sensing. The LOD of anti-DNP sensing was calculated as 1.06 × 10^−9^ g/mL. Chiavaioli et al. [89] developed a fiber-optic sensor with metal oxide nanocoating for precise, femtomolar measurements. The fiber-optic sensor, which used nanocoating-based lossy mode resonance to improve sensitivity, can be potentially used in ultrahigh sensitive immunoassays. Satija et al. [90] explored the use of optimized hollow gold nanoparticles in fiber-optic LSPR sensors to detect goat-anti-human IgG.

As the fiber localized surface plasmon resonance based optical sensor can be used to monitor the interaction between molecules, researchers have studied the molecular binding kinetics by using fiber-optic LSPR sensor for biomedical applications to diagnose or analyze the characteristics of the analytes. Conventional systems such as ”Biacore” products are well known for measuring biomolecular binding kinetics instruments and they are able to measure biomolecular interactions, including protein-protein interactions, or small fragment-protein interactions. Likewise, a monitoring system based on plasmonic nanomaterials to detect the interaction between molecules in real time has been studied in fiber-optic sensors. Chang et al. [91] proposed plasmon resonance based biosensing method for determining the binding kinetic constants of antiovalbumin antibody (anti-OVA) and anti-mouse IgG antibody using fiber-optic particle plasmon resonance biosensor.

**Figure 10:**
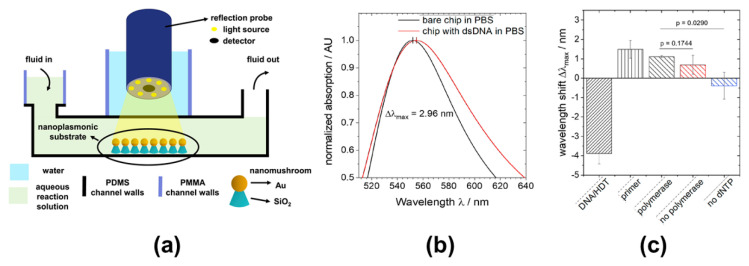
(**a**) Schematic of a DNA polymerase reaction detection platform, which combines a fiber-optic sensor and a microfluidic device with a gold nanomushroom substrate. (**b**) Spectra of bare- and double-stranded 30-mer-DNA-immobilized substrates measured by the fiber-optic-sensor-based polymerase reaction detection platform and (**c**) wavelength shifts according to polymerase reaction steps. The figure in Roether et al. [96] is reprinted with permission from Elsevier.

Deoxyribo nucleic acid (DNA), a biochemical material that serves as the carrier of genetic information for most living things, can provide specific biological information [92]. The modality for measuring data on the target DNA is similar to that of a biological immunosensor based on antibody–antigen interactions. A probe DNA, that can bind to the target DNA, is attached to the sensor and the signal due to the probe–target-DNA binding is measured [93,94]. Sensors measuring DNA and its related interactions require a higher sensitivity enhancement because DNA has a smaller size than antigens and antibodies. Extensive research has been conducted on fiber-optic LSPR sensors using nanomaterials for DNA detection. Kaye et al. [95] proposed a compact fiber-optic LSPR nanoprobe for label-free and sensitivity-enhanced detection of DNA hybridization. Gold nano-posts with a height of 55 nm and a width of approximately 180 nm fabricated at the end of the optical fiber served as the nanoprobe. The fiber-optic nanoprobe with highly enhanced sensitivity could measure probe-target Archaea DNA binding (10 fM of DNA with 20 bases). Roether et al. [96] investigated a DNA polymerase reaction detection platform, which combines a fiber-optic sensor and a microfluidic device with a gold nanomushroom substrate as illustrated in Figure 10. Each nanomushroom consisted of a gold cap with a radius of 11.1 ± 5.2 nm and a bottom pillar made of silicone dioxide (SiO_2_). The DNA polymerase reaction was measured by the optical fiber sensor located at the top of the nanomushroom substrate in the microfluidic device. As described in Figure 10b,c, the platform enabled the measurements and monitoring of DNA polymerase reaction processes with high sensitivity as LSPR was produced by the nanomushroom substrate.

**Figure 11:**
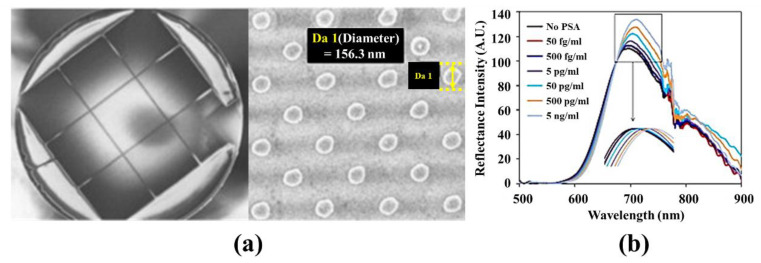
(**a**) Image of the end face of a miniaturized fiber-optic nanoprobe for label-free and highly-sensitive detection of free PSA (f-PSA) and a scanning electron microscopic image of gold nanodisks on the fiber-optic f-PSA sensor. (**b**) Reflectance intensities measured by the fiber-optic f-PSA sensor with varying concentrations of f-PSA. The reprint of figures in Sanders et al. [101] was permitted by Elsevier.

Several studies have shown that a fiber-optic LSPR biological sensor with nanomaterials can be employed for in vitro biomedical diagnosis of specific diseases. Prostate specific antigen (PSA), a glycoprotein enzyme produced from prostate epithelial cells, is one of the biomedical markers for prostate disorders such as prostate cancer [97,98]. Electrochemiluminescence immunoassay are generally employed for measuring PSA concentrations in clinical diagnosis [99,100]. Several research groups have investigated fiber-optic LSPR sensors for label-free and highly-sensitive PSA concentration measurements. Sanders et al. developed a miniaturized fiber-optic nanoprobe for label-free and highly-sensitive detection of free PSA (f-PSA), as illustrated in Figure 11 [101]. At the end of the nanoprobe, 75 × 75 gold nanodisks were fabricated with a period of 400 nm, and a biochemical probe for specific detection of f-PSA was immobilized with a SAM as described in Figure 11a [101]. Control experiments using f-PSA solutions with different concentrations confirmed that the fiber-optic nanoprobe has an LOD of 100 fg/mL. Kim et al. [102] proposed a highly sensitive, real-time PSA measurement platform by combining a fiber-optic LSPR sensor with a microfluidic device. The end of the fiber-optic LSPR sensor with immobilized gold nanoparticles was installed on the side of an outlet in the microfluidic device. The microfluidic device was configured to allow the entire PSA detection process (from the immobilization of a biochemical PSA indicator to the measurement of PSA concentrations in specimens) to be performed in the device. A follow-up study on establishing the nanostructure fabrication process and applying regularly fabricated gold nanodisk-based fiber-optic LSPR sensors to the microfluidic device for highly-sensitive detection of PSA was also reported [103].

**Figure 12:**
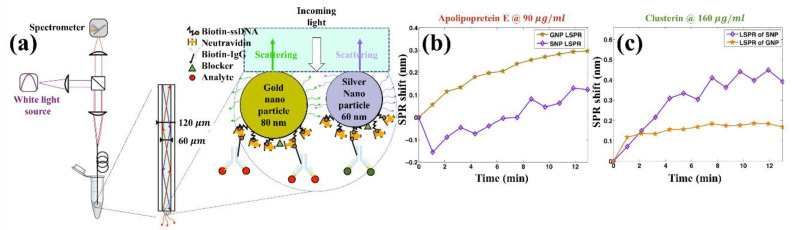
(**a**) Schematic of a fiber-optic LSPR biological sensor that integrates two different types of nanoparticles (gold nanoparticles with a diameter of 80 nm and silver nanoparticles with a diameter of 60 nm) for two different gastric cancer biomarkers. (**b**) The localized surface plasmon resonance (LSPR) was found to shift when the fiber-optic sensor was exposed to a solution of apolipoprotein E with a concentration of 90 μg/mL. Apolipoprotein E was detectable by gold nanoparticles in the fiber-optic sensor with a linear response. (**c**) The LSPR was found to shift when the fiber-optic sensor was exposed to a solution of clusterin with a concentration of 160 μg/mL. Clusterin was measurable by silver nanoparticles in the fiber-optic sensor. The figure in Sciacca et al. [104] is reprinted with permission from ACS Publications, Copyright (2020) American Chemical Society.

A study was conducted on the detection of multiple biomarkers using a single fiber-optic LSPR probe. Sciacca and Monro proposed a fiber-optic LSPR biological sensor that integrated two different types of nanoparticles (gold nanoparticles with a diameter of 80 nm and silver nanoparticles with a diameter of 60 nm) for two different antibodies as illustrated in Figure 12 [104]. As the optical properties of gold and silver are different, the fiber-optic LSPR probe with the gold and silver nanoparticles could detect different antigens in a single spectral measurement system when different antigens are attached to each type of nanoparticle. A preliminary study for detecting two different gastric cancer biomarkers (apolipoprotein E and clusterin) confirmed that this fiber-optic LSPR probe with immobilized gold and silver nanoparticles could be applied for in vitro diagnosis and biochemical analysis.

Fiber-optic LSPR sensors based on nanomaterials can also be used as in vitro diagnostic sensors of infectious diseases. Camara et al. developed a fiber-optic LSPR sensor for the diagnosis of dengue fever [105]. The fiber-optic LSPR dengue immunosensor was fabricated by annealing a gold film (thickness = 6 nm) coated on the end of an optical fiber and immobilizing dengue anti-NS1 (nonstructural protein 1) antibodies after ligand depositions. Preliminary tests to detect dengue NS1 antigen confirmed that the developed fiber-optic LSPR dengue sensor has a limit of quantification of 0.074 μg/mL.

In addition to biomarkers that indicate a specific disease directly, fiber-optic LSPR biological sensors for the detection of health status based on various chemical factors have been actively developed. Semwal and Gupta developed a fiber-optic LSPR sensor to detect cholesterol concentration in specimens [106]. The fiber-optic LSPR cholesterol sensor consisted of a portion of the optical fiber core exposed on the side. The exposed fiber core is deposited with a silver nanofilm, a graphene oxide nanosheet, silver nanoparticles, and an enzyme (cholesterol oxidase) that can absorb cholesterol. In a comparative study of the applicability of the graphene oxide nanosheet and the silver nanoparticles, the fiber-optic cholesterol nanoprobe with both the nanosheet and the nanoparticles had the highest sensitivity for detecting cholesterol (LOD = 1.131 mM). Raj et al. developed a fiber-optic LSPR sensor using silver nanoparticles for measuring the concentration of dopamine, one of the most important neurotransmitters (LOD = 2 × 10^−7^ M) [107]. Khan et al. [108] proposed a fiber-optic Fabry-Perot interferometry sensor with coating of pH-sensitive and solvatochromic dyes for the detection of both pH and glucose concentrations. The developed fiber-optic glucose sensor with a gold nanoparticle-coated tip offers a wider dynamic range (1 μM–1 M) and a highly enhanced sensitivity (3.25 nm/mM) for glucose sensing.

**Figure 13:**
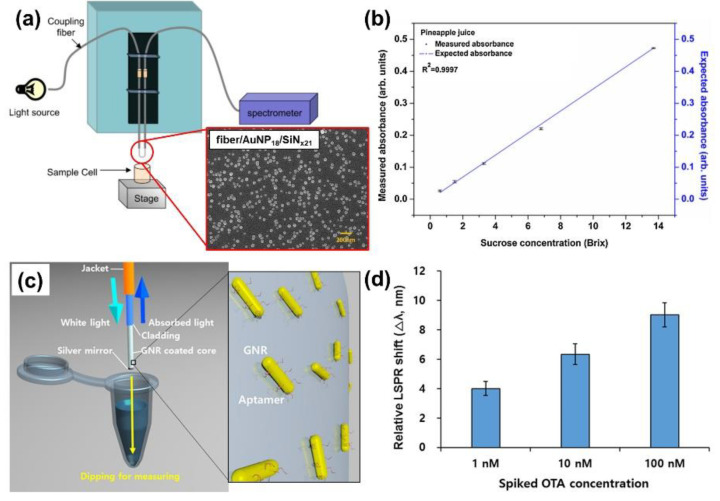
Fiber-optic LSPR sensors for food safety and assessment. (**a**) Schematic of a fiber-optic probe integrated with gold nanoparticles deposited on a silicon nitride substrate for measuring sucrose (sugar) content in fruit juices. (**b**) Absorbance of various sucrose concentrations in commercial pineapple juice measured by the fiber-optic LSPR sensor. The figures in Chauhan et al. [113] are reprinted with permission from Elsevier. (**c**) Schematic of a fiber-optic LSPR sensor based on the integration of colloidal, aptamer-modified nanoparticles for the measurement of ochratoxin A. (**d**) Spectral shifts of LSPR peak wavelengths with different concentrations of ochratoxin A in 50% grape juice. The reprint of the figures in Lee et al. [115] was permitted by Elsevier.

Ensuring food safety and verifying it scientifically are important requirements in the food industry [109,110,111]. Accordingly, devices and technologies that can ensure food safety are being actively developed. Fiber-optic LSPR sensors based on nanotechnology have also been applied for food safety testing. For detecting ascorbic acid (vitamin C) concentrations in specimens, Shrivastav et al. [112] investigated a fiber-optic LSPR probe combined with a polyaniline-silver nanocomposite layer. The sensor achieved sensitivity improvement along with cost reduction and measurement response time improvement. The fiber-optic LSPR ascorbic acid sensor has the potential to be applied to food/drug component/safety analysis. Chauhan et al. [113] developed a fiber-optic probe integrated with gold nanoparticles deposited on a silicon nitride substrate for measuring the sucrose (sugar) content in fruit juices, as shown in Figure 13a,b. Parametric studies confirmed that the fiber-optic sucrose sensor using both the gold nanoparticles and silicon nitride film (thickness = 20 nm) have higher sugar content measurement sensitivity than the sensor using only gold nanoparticles. In a preliminary study of the practical applications in food contents analysis, the sugar contents in three different commercial juices were measured (Figure 13b), and it was confirmed that the sensor has the potential to be applied to the analysis of ingredients for food or beverages. Chang et al. [114] developed a fiber-optic LSPR colorimetric sensor to detect melamine in milk samples. The fiber-optic melamine sensor was based on the sensitivity improvement by the LSPR derived from unmodified gold nanoparticles. Under optimized conditions, the fiber-optic melamine sensor had an LOD of 33 nM and is expected to be utilized in the development of safety assessment systems capable of detecting the contamination of melamine in various dairy products. A fiber-optic LSPR sensor based on the integration of colloidal, aptamer-modified nanoparticles was investigated for highly sensitive measurements of ochratoxin A, a mycotoxin that contaminates food, as illustrated in Figure 13c,d. Sharma and Gupta [115] developed a fiber-optic LSPR sensor using graphene nano-substrates and Tin(IV) oxide (SnO_2_) nanoparticles for measuring hexachlorobenzene, a disinfectant used in seed treatment but currently prohibited from use [116]. Moreover, a fiber-optic LSPR sensor deposited with a silver nanoscale film, silver nanoparticles, and the target material (tetracycline, one of the antibiotics) on a partially exposed area of the optical fiber was developed [117]. The fiber-optic LSPR tetracycline sensor can detect tetracycline in a wide concentration range (10^−8^–10^−5^ M) and has the potential to be employed to measure the tetracycline concentration in foods.

## 4. Conclusions

In this article, we explored both the fundamentals and the recent applications of fiber-optic LSPR sensors using nanomaterials. The applications of fiber-optic LSPR sensors have been intensively studied in the following three areas: detection of chemical substances in a liquid or gas, measurement of physical properties, and detection of specific biological targets from small amounts of biomaterials as indicators in medical and food fields. Through improved sensitivity based on the fields localized by nanostructures or nanoparticles on the fiber-optic probe, fiber-optic LSPR sensors have the potential to be used in the practical detection of various analytes. Moreover, fiber-optic LSPR sensors can be manufactured in smaller sizes than conventional optical sensors, and they can be applied with minimal invasiveness to various samples from gases to solids. Moreover, the performance comparison results of the fiber-optic sensors with varied localized surface plasmon resonance were investigated. Those comparison results need to be analyzed carefully because the new attempt for dedicated target measurements does not have a direct proportional relationship with performance improvement. Specifically, a new attempt of treatment in fiber with various materials to give a specific response to a binding target may cause some degradation of the sensor’s sensitivity, and the sensitivity is mainly affected by the morphological characterization of nanoparticle/nanostructure and the type of the targets/analytes. Thus, a simple comparison and listing of the results based on the limit-of-detection and listing the degree of improvement in electromagnetic field enhancement were discussed. In chemical fiber-optic localized surface plasmon resonance sensors, Polley et al. [53] reported the holes array-based fiber-optic plasmonic sensors with sensitivity of (420 ±  83) nm/RIU. Pathak and Gupta [54] reported a fiber-optic sensor with a dynamic range of 0–150 μM and a detection limit of 2.1 μM utilizing a carbon nanotube-based SPR technique. A performance in most of fiber-optic LSPR sensors for biological applications is represented in terms of refractive index unit (RIU). Shao et al. [86] indicated the results of sensitivities are 13.09 AU/RIU (R = 0.9678) for 48-nm gold nanoparticles assembled film and 5.85 AU/RIU (R = 0.9666) for 23-nm gold nanoparticles assembled film. Lin et al. [88] reported DNP-functionalized tapered fiber LSPR sensor which shows the refractive index resolution based on the interrogation of transmission intensity change which is calculated to be 3.2 × 10^−5^ RIU. Sanders et al. [101] reported a fiber-optic nanoprobe for sensitive detection of f-PSA, in which the fiber-optic nanoprobe has an LOD of 100 fg/mL. Semwal and Gupta [106] developed a fiber-optic LSPR sensor to detect cholesterol concentration in specimens, with both the nanosheet and the nanoparticles for detecting cholesterol (LOD = 1.131 mM). Raj et al. [107] developed a fiber-optic LSPR sensor using silver nanoparticles for measuring the concentration of dopamine, (LOD = 2 × 10^−7^ M). Chang et al. [114] reported a fiber-optic LSPR colorimetric sensor to detect melamine in milk samples which had an LOD of 33 nM. In the investigation of sensitivity in fiber-optic LSPR sensors using nanostructures or nanoparticles, an optimization of nanomaterials implemented in the fiber-optic LSPR sensor is needed with reflecting the characteristics of optical fiber materials, light sources, and measurement targets. We expected that preemptive exploration using a simulation platform of optical fields on nanomaterials is helpful to optimizing sensitivity of fiber-optic LSPR sensors before experimental developments.

**Figure 14:**
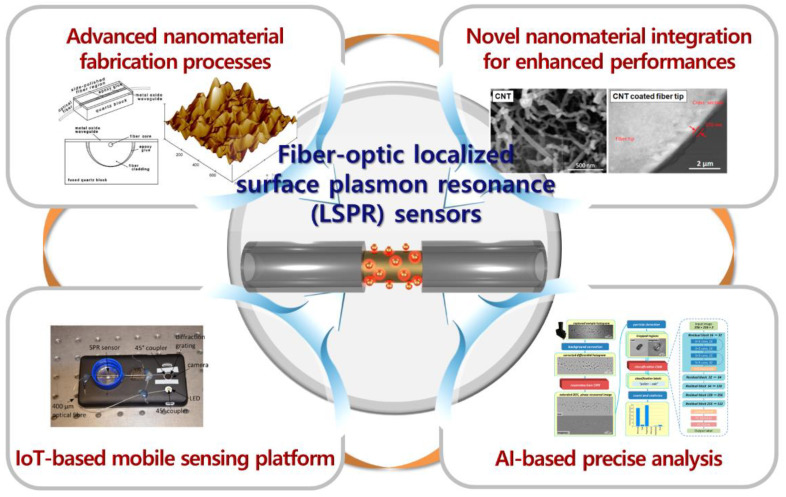
Technologies to enhance the performance of fiber-optic LSPR sensors through further developments and applications. (1) Advanced nanomaterial fabrication processes to ensure uniformity of the plasmonic nanomaterials. (2) Integration of novel nanomaterials to enhance the sensing performance. (3) Application of communication/IoT techniques to produce mobile sensing platforms. (4) Precise analysis by machine learning and AI. The reuse of the inset figures representing each technology was permitted by Elsevier [118], Molecular Diversity Preservation International [119], Optical Society of America [120], and American Chemical Society [121].

Further developments and applications (as described in Figure 14) are required in the following areas to translate the advantages of fiber-optic LSPR sensors into the actual commercialization and production stages.

The variations in the size and morphology of the nanomaterials lead to a large degree of variations in their optical properties. Hence, a high degree of uniformity should be maintained during the process of forming nanomaterials and integrating them on the fiber-optic sensor, and sufficient quality control is also required. Advanced fabrication techniques to produce large-area nanoscale substrates can be employed for enhancing uniformity of nanomaterials on the fiber-optic LSPR sensor [118,122,123]. Further developments are needed to actively employ nanomaterials in fiber-optic LSPR sensors to improve performances. For instance, several research groups have studied carbon nanotube and graphene-based optical sensors to enhance sensitivity or other sensing properties [119,124,125,126]. As it is difficult to use well-aligned light sources and highly sensitive photodetectors in certain practical applications of LSPR fiber-optic sensors, improving the sensing characteristics based on novel nanomaterials is necessary. In addition, it is determined that the integration of advanced communication technologies, which have been applied to various sensors, with fiber-optic LSPR sensors will increase their mobility and usability. For instance, several research groups have introduced optical sensors integrated with a smartphone to form a device that records, stores, transfers, and processes the sensing data [83,120,127]. The combination of Internet-of-Things (IoT) technology with a compact, highly-sensitive fiber-optic sensor is expected to produce an impressive sensor that has chemical, biological, and medical applications. Processing and analyzing data collected through the fiber-optic LSPR sensors to extract meaningful information is also important for various applications. Several recent studies have proposed the integration of photonic sensors and artificial intelligence (AI) [121,128,129]. Data processing and analysis modalities based on AI and machine learning are expected to help enhance the practicality of fiber-optic LSPR sensors.

## Data Availability

No new data were created or analyzed in this study. Data sharing is not applicable to this article.
